# Engineering, Characterization, and Biological Evaluation of an Antibody Targeting the HGF Receptor

**DOI:** 10.3389/fimmu.2021.775151

**Published:** 2021-12-03

**Authors:** Claudia Desole, Simona Gallo, Annapia Vitacolonna, Elisa Vigna, Cristina Basilico, Francesca Montarolo, Francesca Zuppini, Elena Casanova, Riccardo Miggiano, Davide Maria Ferraris, Antonio Bertolotto, Paolo Maria Comoglio, Tiziana Crepaldi

**Affiliations:** ^1^ Department of Oncology, University of Turin, Candiolo, Italy; ^2^ Candiolo Cancer Institute, FPO-IRCCS, Candiolo, Italy; ^3^ Neuroscience Institute Cavalieri Ottolenghi (NICO), Orbassano, Italy; ^4^ Department of Molecular Biotechnology and Health Sciences, University of Turin, Torino, Italy; ^5^ Department of Pharmaceutical Sciences, University of Piemonte Orientale, Novara, Italy; ^6^ IXTAL srl, Novara, Italy; ^7^ IFOM, FIRC Institute for Molecular Oncology, Milano, Italy

**Keywords:** HGF, MET, PSI domains, antibody engineering, regenerative medicine, anti-cancer therapy, multiple sclerosis, experimental autoimmune encephalomyelitis

## Abstract

The Hepatocyte growth factor (HGF) and its receptor (MET) promote several physiological activities such as tissue regeneration and protection from cell injury of epithelial, endothelial, neuronal and muscle cells. The therapeutic potential of MET activation has been scrutinized in the treatment of acute tissue injury, chronic inflammation, such as renal fibrosis and multiple sclerosis (MS), cardiovascular and neurodegenerative diseases. On the other hand, the HGF-MET signaling pathway may be caught by cancer cells and turned to work for invasion, metastasis, and drug resistance in the tumor microenvironment. Here, we engineered a recombinant antibody (RDO24) and two derived fragments, binding the extracellular domain (ECD) of the MET protein. The antibody binds with high affinity (8 nM) to MET ECD and does not cross-react with the closely related receptors RON nor with Semaphorin 4D. Deletion mapping studies and computational modeling show that RDO24 binds to the structure bent on the Plexin-Semaphorin-Integrin (PSI) domain, implicating the PSI domain in its binding to MET. The intact RDO24 antibody and the bivalent Fab2, but not the monovalent Fab induce MET auto-phosphorylation, mimicking the mechanism of action of HGF that activates the receptor by dimerization. Accordingly, the bivalent recombinant molecules induce HGF biological responses, such as cell migration and wound healing, behaving as MET agonists of therapeutic interest in regenerative medicine. *In vivo* administration of RDO24 in the murine model of MS, represented by experimental autoimmune encephalomyelitis (EAE), delays the EAE onset, mitigates the early clinical symptoms, and reduces inflammatory infiltrates. Altogether, these results suggest that engineered RDO24 antibody may be beneficial in multiple sclerosis and possibly other types of inflammatory disorders.

## Introduction

Hepatocyte growth factor (HGF), also known as scatter factor, is a multi-functional cytokine that was originally cloned as a potent mitogen for hepatocytes in primary cultures (1) and was later identified as a cell motility factor for epithelial, muscle and nerve cells (2). HGF is produced and secreted as an inactive pro-HGF form (92 kDa) by stroma cells such as macrophages and fibroblasts. Secreted pro-HGF is cleaved between Arg494 and Val495 to obtain active HGF, which is constituted by two chains, covalently linked by a disulfide bridge: a heavy chain of 62 kDa and a light-chain of 32-36 kDa. The activation of HGF is mediated by various proteases such as urokinase, plasminogen activator, and HGF activator (3, 4). The latter is the most efficient and is itself activated upon thrombin cleavage of its precursor (5). HGF is the ligand of the MET receptor tyrosine kinase (6, 7). The biologically active αβ heterodimeric HGF contains a high affinity MET-binding site in the α chain (8) and a low-affinity MET-binding site in the β chain (9). Only the active form of HGF is able to elicit MET-mediated biological activities.

MET receptor is synthesized as a single-chain precursor that is cleaved into two disulfide bond-linked α and β subunits of 50 and 140 kDa, respectively, by the furin protease in the Golgi apparatus (10). The MET αβ heterodimer consists of an N terminal extracellular domain (MET ECD), a trans-membrane domain, a juxta membrane region, an intracellular tyrosine kinase domain and a C-terminal tail. The MET ECD is composed of a Semaphorin (SEMA) domain, a Plexin-Semaphorin-Integrin domain (PSI), and four Immunoglobulin-like regions found in Plexins and Transcription factor (IPT1-4). The SEMA domain is necessary and sufficient for HGF binding (11) and is required for receptor dimerization and activation (12). The IPT3,4 regions have also been proposed as a further MET binding site for the HGF α-chain (13). HGF binding upon MET activates the receptor leading to several biological responses including cell scattering, motility, survival and differentiation. MET is primarily expressed in epithelial and endothelial cells (14), but also in some myoblasts and neuronal precursors thus contributing to the development of muscular and nervous structures (15–18). The normal expression and function of MET and HGF are fundamental during embryogenesis, promoting growth and development of hepatocytes, placental trophoblasts and myoblasts (16, 18, 19). Furthermore, MET activation is involved in organ growth and after injury regeneration, angiogenesis, wound healing, scattering, and proliferation (20–22).

Thus, MET activation is an interesting target in regenerative medicine and HGF role has been investigated in a panel of injury/disease models (23). The use of MET-deleted mice demonstrated the involvement of HGF and MET in regeneration, protection, and homeostasis of tissues. Many studies demonstrated that HGF is a powerful neurotrophic factor in the nervous system with beneficial and protective effects in various animal disease models. Thus, a possible therapeutic application of HGF is suggested for the treatment of neurological, neurodegenerative, and psychiatric disorders (see Desole et al., 2021 for a recent review) (24). HGF and MET play also important cardioprotective roles in the injured heart, by promoting pro-survival effects in cardiomyocytes, such as protection from apoptosis, autophagy, and genotoxicity (25, 26). Moreover, HGF drives migration and proliferation of cardiac stem cells (27, 28). Furthermore, studies on MET-deleted mice revealed a fundamental inhibitory role of HGF in the progression of chronic inflammation and fibrosis. Chronic tissue injury and inflammation have been associated with the onset of fibrosis. HGF treatment resulted in reduced fibrosis and improved tissue functions in a panel of disease models, such as liver cirrhosis, chronic kidney disease, and chronic fibrosis, due to its anti-apoptotic and anti-inflammatory activity (29–37). In the experimental autoimmune encephalomyelitis (EAE) model of multiple sclerosis (MS), mesenchymal stem cells transplantation ameliorated EAE clinical symptoms and this benefit is caused in large part by the production of HGF (38).

On the other hand, HGF-MET signaling plays a role in cancer cells, conferring malignant features such as invasion, metastasis, and drug resistance in the tumor microenvironment (22). The oncogenic gain of function of MET is obtained through different mechanisms. Genetic alterations of MET gene, such as gene amplification, point mutations or transcriptional activation, result in constitutive activation of MET kinase, which turns into a powerful oncogenic receptor insensitive to ligand regulation (39). Targeting of constitutively active MET by means of inhibitors is useful to blunt tumor growth in experimental models and in patients (40, 41). Moreover, stressful signals that promote transcriptional activation of MET in cancer cells may also induce HGF upregulation in tumor stromal cells (42), feeding a positive stimulatory circuit of HGF-MET signaling that allows cancer cells to convert the anti-apoptotic and pro-migratory activities typically used in tissue regeneration and repair into pro-invasive and pro-metastatic behaviors (43). Thus, several MET-targeting agents, including HGF and MET antibodies, as well as small molecule kinase inhibitors, are currently envisaged as anti-cancer therapeutics (44).

In a previous work, we generated a mouse monoclonal antibody (mAb), known in the art as DO24 (45), endowed with MET agonist activity (46). We also demonstrated that this agonist mAb protects cardiomyocytes from hypoxic and chemotherapy injury, thus displaying potential therapeutic properties (25, 26). Here, we describe the generation and characterization of a synthetic antibody and antibody fragments derived from DO24 mAb. We recovered and cloned the VH (heavy chain variable domain) and VL (light chain variable domain) cDNA sequences from DO24 mouse hybridoma into appropriate vectors, introduced the vectors into host mammalian cells, and achieved expression of adequate amounts of functional antibody. The recombinant antibody (RDO24_mIgG2a) and its fragments (RDO24_mFab2 and mFab) were thus generated *in vitro* and assessed for MET agonism. Only the bivalent molecules demonstrated agonistic activity. Moreover, binding of recombinant antibody required the PSI and not the SEMA domain of MET, suggesting different molecular mechanisms for receptor activation. The stable full-size RDO24 was tested in the EAE model and showed a delay in the disease onset and severity. These engineered anti-MET molecules are promising candidates for therapeutic purposes and will be useful tools for further exploring MET receptor activation.

## Materials and Methods

### Design, Synthesis, and Purification of the RDO24_mIgG2a, RDO24_mFab2, and RDO24_mFab

Molecules have been designed by inserting the synthetic codon optimized VH and VL cDNA sequences from DO24 mouse mAb in pcDNA3.1 vector which carry the murine constant IgG2a (immunoglobulin 2a) heavy chain (CH1, CH2, CH3) and the murine constant k light chain (CL), respectively. DNA plasmids were produced as endotoxin-free preparations and sequence-verified to confirm identity prior to mammalian ExpiCHO-S transient transfection (FlowEighteen38, Porto, PT). RDO24_mIgG2a proteins were purified using a HiTrapMabSelect Sure Protein A 5 mL column (GE Healthcare, Buckinghamshire, UK) on an ÄKTA Pure 25L FPLC system. RDO24_mFab2 and RDO24_mFab proteins were purified using a HisTrap HP 5 mL column (GE Healthcare). Analysis of the purified molecules was performed by SDSPAGE under reducing and non-reducing conditions, followed by GelCode Blue Stain reagent (Pierce, Waltham, MA). The production of DO24 mAb, the antibody from which RDO24 molecules were derived, was performed as previously described (46). Briefly, DO24 was produced from hybridomas obtained with the fusion between the immune spleen cells from Balb/c mice (Charles River Laboratories, Wilmington, MA, USA) immunized with GTL-16 cells, where the *MET* gene is amplified and overexpressed, and the P3.X63.Ag8.653 myeloma cells.

### Cell Culture and Materials

H9c2, A549, HEK293T, GTL16, TOV-112D, MDCK and HPAF-II cell lines were purchased from the American Type Culture Collection (ATCC, Manassas, VA, USA). Rhabdomyosarcoma cells were kindly provided by Riccardo Taulli (University of Turin). HUVECs were obtained from 15 samples following parental consent, and grown in gelatin-coated plates in their own complete medium (M199 medium supplemented with 10% FCS, 0.02% extract of bovine brain, and 0.015% porcine heparin). A549 and GTL16 cells were cultured in RPMI medium; H9c2 and rhabdomyosarcoma cells were grown in DMEM medium, HEK293T were cultured in Iscove’s medium, and MDCK and HPAF-II in EMEM medium. TOV-112D were cultured using a 1:1 mixture of MCDB 105 medium and medium 199 supplemented with 15% fetal bovine serum (FBS). RPMI, Iscove, DMEM, and EMEM media were supplemented with 10% FBS, 1% penicillin, 1% streptomycin and 1% L-Glutamine. Cells were incubated under 5% CO2 at 37°C, were passed regularly and sub-cultured to ~80/90% of confluence. Unless specified, all materials were from SigmaAldrich (St. Louis, Missouri, USA). Anti-pMET (Y1234/1235; 3077), -MET (D1C2; 8198), -pERK (T202/Y204; 4376), -pAKT (S473; 9271), -pCREB(S133; 9198), and -CREB (4820) antibodies were purchased from Cell Signaling Technology (Danvers, MA, USA), and anti-ERK (C14; SC-154) and -AKT (C-20; SC-1618) from Santa Cruz (Santa Cruz, CA, USA). HGF (Recombinant Human Hepatocyte Growth Factor NS0-expressed) was purchased from R&D systems (Minneapolis, Minnesota, USA).

### Surface Plasmon Resonance

The kinetic constants of RDO24, Fab2, and Fab with recombinant human MET ECD-Fc (R&D) were measured using a Biacore T100 instrument (GE Healthcare) and the CM5 chip, following standard procedures. Human MET ECD-Fc (pH 4) was immobilized onto the surface of a single channel of the CM5 sensor chip by amine coupling. In a single-cycle kinetics experiment, RDO24_mIgG2a, RDO24_mFab2, and RDO24_mFab solutions were separately injected in four flushes at increasing concentrations (from 41 nM to 330 nM for RDO24mIgG2a or from 62 nM to 500 nM for RDO24_mFab and RDO24_mFab2) over the human MET ECD-Fc functionalized sensor surface, using HBS-EP+ (Cytiva) as running buffer, with a contact time of 120 seconds. The reference flow cell was used as a control surface for refractive index change and nonspecific binding. A long dissociation phase (600 seconds) and a single regeneration step followed the last sample injection (NaOH 50 mM, 30 seconds of contact time), without regeneration between each sample injections.

### Flow Cytometry

Human GTL16, A549, HEK293T, HUVEC, rat H9c2, and mouse rhabdomyosarcoma cells were resuspended in PBS 1% FBS and then stained for 15 min at room temperature, in the dark, with RDO24_mIgG2a labeled by PE-Cy7 fluorochrome, using Lightning-Link (Innova Biosciences, Cambridge, UK). To exclude died cells the stained cells were resuspended in Phosphate Buffered Saline (PBS) Dapi 0.2X solution. The negative control was the unstained cells. Samples were analyzed on a CyAn™ ADP LX nine-color analyzer (Beckman Coulter, Brea, CA, USA).

### Lentiviral Vectors and Engineering of MET Wild-Type and MET Deletion Mutants

MET transmembrane receptors described in this work have been generated by standard PCR and genetic engineering techniques. All of the proteins conserve the signal peptide of their parental polypeptide at the Nterminus. The receptors are identical to MET wild-type (WT: total amino-acid length 25-1390), except for the deletion of SEMA domain (MET ΔSEMA: Δ25-516) or SEMA and PSI domains (MET ΔSEMA-PSI: Δ25-562) or IPT domains (MET ΔIPT: Δ563-932) or PSI and IPT domains (MET ΔPSI-IPT: Δ517-932). The cDNAs encoding all of the engineered proteins were subcloned into the lentiviral transfer vector pRRL2. Vector stocks were produced as previously described (47). Viral p24 antigen concentration was determined by the human immunodeficiency virus, type 1 p24 core profile ELISA kit (PerkinElmer Life Sciences, Waltham, MA, USA) according to the manufacturer’s instructions. Human ovarian carcinoma TOV-112D cells (that do not express MET endogenously) were seeded into a 6wells plate (50000 cells/well in 2 mL medium). The following day cells were washed with PBS and transduced using 100 ng/ml or 500 ng/ml of p24 in the presence of 0.1% polybrene (Sigma-Aldrich). Cells were incubated for 24 h, then media were changed and cells were grown to confluence.

### Immunoprecipitation Assay

TOV-112D cells without or with ectopic expression of MET WT or MET deletion mutants were lysed with cold RIPA buffer in the presence of 1 mM Na3VO4 and a cocktail of protease inhibitors (all from Sigma-Aldrich). Total protein lysates were incubated at 4°C overnight on rotor with RDO24 mAb, then Sepharose-protein A (GE Healthcare) was added and the samples were incubated for additional 2 h at 4°C. As control, an equal amount of total proteins was incubated with Sepharose protein A in the absence of antibodies. After five washes with cold RIPA buffer, immunoprecipitated proteins were eluted with boiling Laemly buffer and analyzed by western blotting.

### Enzyme-Linked Immunosorbent Assays

To analyze the specificity and selectivity of RDO24_mIgG2a, 96well EIA/RIA plates (Costar, #3590, Corning, NY, USA) were coated at 4°C overnight with 100ng/well recombinant Fc-fused extracellular domain-fragment (ECD-Fc) of human MET (Recombinant Human HGFR/c-MET Fc Chimera His-tag Protein, R&D) or RON ECD (Recombinant Human MSP R/RON, R&D) or SEMA4D ECD-Fc (Recombinant Human Semaphorin4D Fc Chimera Protein, R&D). Following three washes with PBS with 0.05% Tween 20, they were blocked with PBS-0.5% BSA (bovine serum albumin) for 1 h at 37°C. Wells were then washed thrice with PBS-0.05% Tween and then RDO24_mIgG2a or anti-RON (RON monoclonal antibody, MA5-31073, Invitrogen) or antiSEMA4D (SEMA4D monoclonal antibody, eBio133-1C6, Invitrogen) were incubated at increasing concentration (0-100 nM) at 4°C overnight. After three washes with PBS with 0.05% Tween 20, wells were incubated with 1:5000 mouse horseradish peroxidase-conjugated secondary antibody (Jackson Laboratory, Bar Harbor, ME, USA) for 1 h at room temperature, followed by three more washes with PBS with 0.05% Tween 20. Tetramethylbenzidine (TMB) substrate solution (100 µl/well) was then added and quenched after 3-4 minutes with 25 µL of 2 M H_2_SO_4_. Colorimetric assay was quantified by the multi-label plate reader VICTOR-X4 (Perkin Elmer Instruments INC.). Binding data were analyzed and fitted using Prism software (Graph Pad Software, San Diego, CA, USA).

### Protein Modeling and Docking

The sequences of the VH and VL segment of RDO24 were assembled in the Fv format by homology modeling using the Rosetta-based computational homology modeling technique (48). The RDO24/MET docking model was made using the ZDOCK server (49). Structure models were analyzed using Discovery Studio Visualizer.

### Western Blot Analyses

A549 cells were cultured in low serum medium (0.5% FBS) for 24 h and then treated. For dose-dependent analysis HGF (1 nM), RDO24 mIgG2a (125 nM), mFab2 (125 nM) and mFab (125 nM) were administered for five minutes. For time course analysis DO24 mAb and RDO24 molecules were used at 50 nM and HGF at 0.6 nM. After the treatment, the cells were lysed in ice-cold RIPA lysis buffer added with protease inhibitor cocktail (Sigma-Aldrich). Lysates were subsequently sonicated and centrifuged at 12,000 g at +4°C (20 min). The protein concentration was evaluated with the BCA Protein Assay Kit (Thermo Fisher Scientific, Waltham, MA, USA). Proteins and pre-stained protein ladder (10-180 kDa, PageRuler™, Thermo Fisher Scientific) were separated by electrophoresis using precast 4-12% SDS-PAGE gels (Invitrogen, Carlsbad, CA, USA) and transferred to Hybond-P pvdf membrane (Bio-Rad, Hercules, CA, USA). After incubation in blocking solution (10% bovine serum albumin, BSA, Sigma-Aldrich) at room temperature, membranes were incubated overnight at +4°C with the primary antibodies. Primary antibodies were diluted in BSA 5% TBS (tris-buffered saline) Tween and re-used at most three times. α-tubulin (Sigma-Aldrich) was used as loading control. Bands obtained from the same blot as the target protein of interest were exploited for normalization. Membranes were washed and then incubated with specific horseradish peroxidase-conjugated secondary antibodies (Jackson Laboratory) for 1 h at room temperature. Secondary antibodies were diluted in TBS Tween and used once only. The proteins were revealed by enhanced chemiluminescence of the ECL Prime detection kit (Bio-Rad) and quantified with the Image Lab software (Bio-Rad). Quantitation of band density was conducted on analysis within the linear range and by a blinding approach.

### Scatter and XCelligence Assays

For end-point analysis, MDCK cells (12000 cells/well) were seeded in 96-well plates in complete culture medium. After 24 h, cells were incubated in the presence of RDO24_mIgG2a, mFab2 and mFab (50 nM), or HGF (0.5 nM) for 20 h. Cells were fixed with 11% glutaraldehyde and stained with 0.1% Crystal Violet (SigmaAldrich). For real-time cell motility assay, HPAF-II cells (10000 cells/well) were seeded in E-plates (Roche Diagnostics, Mannheim, Germany) in complete culture medium and treated as above. Electrical impedance was monitored continuously for 24 h using an X-Celligence RTCA device (Roche Diagnostic). The electronic readout of cell-sensor impedance is displayed in real-time as cell index, a value directly influenced by cell shape and spreading. The induction of cell flattening and cell dissociation results in an increase of the cell index.

### Wound Healing Assay

H9c2 cardiomyoblasts (150000/well) were plated in 24-well plates and maintained in DMEM 10% FBS until confluence, and then were incubated in DMEM 0.5% FBS for 18 h. A scratch wound was made by scratching with a 10-μL pipette tip across the center of the well. Then cells were washed with PBS, left untreated or incubated for 24 h with HGF, whole RDO24 mAb, and fragments. Images of wound at the start moment and after the treatment were taken with DMRI Leica inverted microscope. Migration was quantified by evaluating the area of wound at time zero (A0) and at time after the treatment (Ay = 24 h). Normalization and quantification on the basis of three independent experiments were obtained by the formula (A0−Ay)/A0.

### Animals

All experimental procedures were carried out at Neuroscience Institute Cavalieri Ottolenghi (NICO), approved by the Ethical Committee of the University of Torino, and authorized by the Italian Ministry of Health (authorization number: 168/2020-PR). The experiments were performed in accordance with the European Community Parliament and Council Directives of 24 November 1986 (86/609/EEC) and 22 September 2010 (2010/63/EU). Mice were housed with a 12 h light/dark cycle and free access to food/water. Adequate measures were taken to minimize pain and discomfort. Female C57BL/6J mice used for all the experimental procedures were purchased from Envigo RMS srl (Udine, Italy).

### Antibody Plasma Concentration Kinetic

RDO24_mIgG2a, RDO24_mFab2, or RDO24_mFab were intravenously injected in C57BL/6J mice (n=3) at the following concentration 10, 7 and 3.5 mg/kg, respectively. Plasma was collected after 1, 6, 24, 72 and 120 hours for each mouse and diluted 1:800 in PBS for antibodies quantification. 96well EIA/RIA plates (Costar) were coated at 4°C overnight with 100ng/well human MET ECD-Fc (R&D). Following three washes with PBS with 0.05% Tween 20, they were blocked with PBS-0.5% BSA for 1 h at 37°C. Wells were then washed thrice with PBS-0.05% Tween and then plasma were incubated at 4°C overnight. After three washes with PBS with 0.05% Tween 20, wells were incubated with 1:5000 mouse horseradish peroxidase-conjugated secondary antibody (Jackson Laboratory) for 1 h at room temperature, followed by three more washes with PBS with 0.05% Tween 20. TMB substrate solution (100 µl/well) was then added and quenched after 3-4 minutes with 25 µL of 2 M H_2_SO_4_. Colorimetric assay was quantified by the multi-label plate reader VICTOR-X4 (Perkin Elmer Instruments INC.).

### EAE Induction and Clinical Evaluation

As reported in Montarolo et al., 2014, 2015, 2021 (50–52), to induce EAE, 6–8 week-old-female C57BL/6 mice were immunized by subcutaneous injection under the rostral part of the flanks and at the base of the tail with 300 µl of 200 µg/mouse of myelin oligodendrocyte glycoprotein (MOG_35–55_; Espikem, Florence, Italy) in incomplete Freund’s adjuvant (IFA; Sigma-Aldrich, Milan, Italy) containing 8 mg/mL Mycobacterium tuberculosis (strain H37Ra; Difco Laboratories Inc., Franklin Lakes, NJ, USA). Mice were treated with two intravenous injections of 500 ng of Pertussis toxin (Duotech, Milan, Italy) on the immunization day and 48 h later. Clinical score (0 = healthy; 1 = limp tail; 2 = ataxia and/or paresis of hind limbs; 3 = paralysis of hind limbs and/or paresis of forelimbs; 4 = tetraplegia; 5 = moribund or dead) was recorded daily by an investigator blind to group identity. The percentage of disease-free mice was calculated evaluating the day post immunization (dpi) when the first clinical manifestations appeared (score>0). Cumulative score was calculated as the sum of the daily score during experiment. To obtain the final concentration (10 mg/kg) RD024 was dissolved in saline solution (0.9% NaCl). Control animals received the vehicle (0.9% NaCl, vehicle). The RD024 or vehicle EAE mice were intravenously injected at 6, 8, and 10 days post immunization (dpi). The experiment with 5 vehicle- and 5 RD024-treated animals was twice repeated.

### Histological Evaluation

EAE mice were deeply anesthetized (zoletil 100 mg/kg, xylazina 5 mg/kg) and trans-cardially perfused with 4% paraformaldehyde in 0.12 M phosphate buffer, pH 7.2–7.4. The spinal cords were removed and immersed in the same fixative at 4°C for 24 h and then cryo-protected in 30% sucrose in 0.12 M phosphate buffer. Spinal cord were frozen and serially cut by a cryostat in 30 μm-thick coronal sections collected in PBS. For immunofluorescence, sections were stained to detect the expression of MOG (1:200, Proteintech, Rosemont, IL, USA) antigen. Incubation with primary antibody was made overnight at 4°C in PBS with 0.5% Triton-X 100. The sections were then exposed for 2 h at room temperature with secondary Alexa Fluor 555 (Molecular Probes Inc, Eugene, regon) -conjugated antibody. 4,6-diamidino-2-phenylindole (DAPI, Fluka, Saint Louis, USA) was used to counterstain cell nuclei. After processing, sections were mounted on microscope slides with Tris-glycerol supplemented with 10% Mowiol (Calbiochem, LaJolla, CA). Histological specimens were examined using ZEISS Axioscan 7 Microscope Slide Scanner (Weltzar, Germany). Adobe Photoshop 6.0 (Adobe Systems, San Jose, CA) was used to assemble the final plates. Quantitative evaluations were performed on images followed by ImageJ (Research Service Branch, National Institutes of Health, Bethesda, MD; available at http://rsb.info.nih.gov/ij/) analyses. The expression level of MOG staining as positive fractioned area (i.e. the percentage of positive pixels throughout the entire area) was quantified to analyse the demyelinated areas. Inflammatory infiltrates were assessed using DAPI staining.

### Statistical Analysis

All values are expressed as the mean ± standard deviation of, at least, 3 independent experiments. T-test was used to statistically compare two groups. For data representation in graph we performed data transformation expressing the values as ‘fold mean control’. For animal studies data were represented as mean ± standard error (SE) or median. Normality of distribution was assessed by the Shapiro-Wilk test. Two-way ANOVA followed by the Bonferroni’s multiple comparison *post-hoc* test was used to compare the clinical score during days. Mann–Whitney U test was used to compare continuous data between groups. Chi-square test was used to compare the onset of EAE. In all instances, the threshold P value deemed to constitute statistical significance was <0.05. The data analysis and the graph design were done using GraphPad Prism software.

## Results

### Engineering of the RDO24_mIgG2a, RDO24_mFab2, and RDO24_mFab

**Figure 1:**
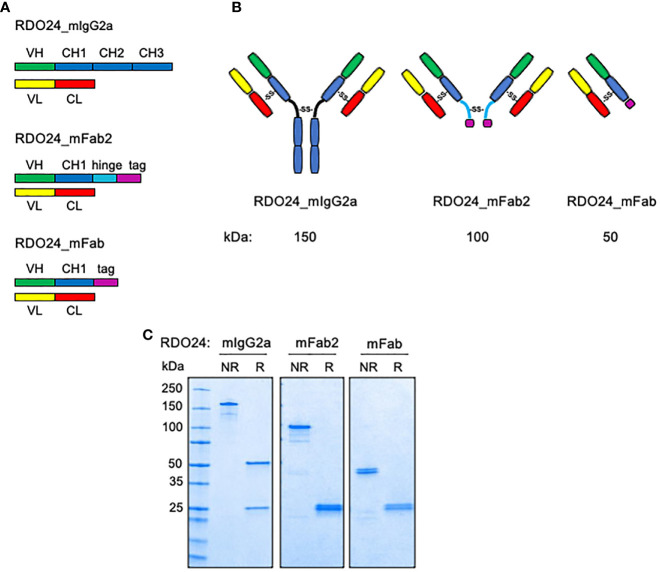
Schematic representation **(A)**, hypothesized structure **(B)**, and western blot analysis **(C)** of RDO24_mIgG2a, RDO24_mFab2 and RDO24_mFab. NR, not-reduced; R, reduced.

We engineered the full-length form of mouse DO24 mAb and its derived recombinant fragments (Fab2 and Fab) (**Figure 1A**). The mAb cDNA was cloned from total RNA of the corresponding hybridoma using RT-PCR (reverse transcriptase-polymerase chain reaction) based on a degenerated oligonucleotide strategy. Then, using the mAb heavy and light chains as templates, the regions corresponding to the variable (VH and VL) domains, respectively, were PCR-amplified. Synthetic codon-optimized gene sequences for VH and VL chains were sub-cloned into relevant pcDNA3.1 vector expressing the murine constant IgG2a heavy chain (CH1, CH2, CH3) or the murine constant k light chain (CL), respectively. The recombinant Fab2 mAb was built by joining the VH-CH1 with the hinge sequence present on the vector, containing the cysteine residues required for dimerization. The recombinant Fab was obtained by expressing VH-CH1, VL-CL sequences. Moreover, the heavy chains of Fab and Fab2 fragments were tagged with a histidine tail for purification purposes. **Figure 1B** shows the drawing of the bivalent and monovalent structures of the molecules. The **Figure 1C** shows that purified recombinant proteins, analyzed under non-reducing conditions, formed covalently linked complexes of 150 kDa (full-length RDO24_IgG2a), 100 kDa (RDO24_mFab2), and 50 kDa (RDO24_mFab). When analyzed under reducing conditions, the full-length antibody showed two bands of 50 and 25 kDa corresponding to the heavy and light chains, respectively. The mFab2 and mFab showed the expected molecular weights of 25 kDa for the shorter heavy chains as well as the light chains.

### RDO24 Antibody and Fragments Bind to the ECD of Human MET With High Affinity

**Figure 2:**
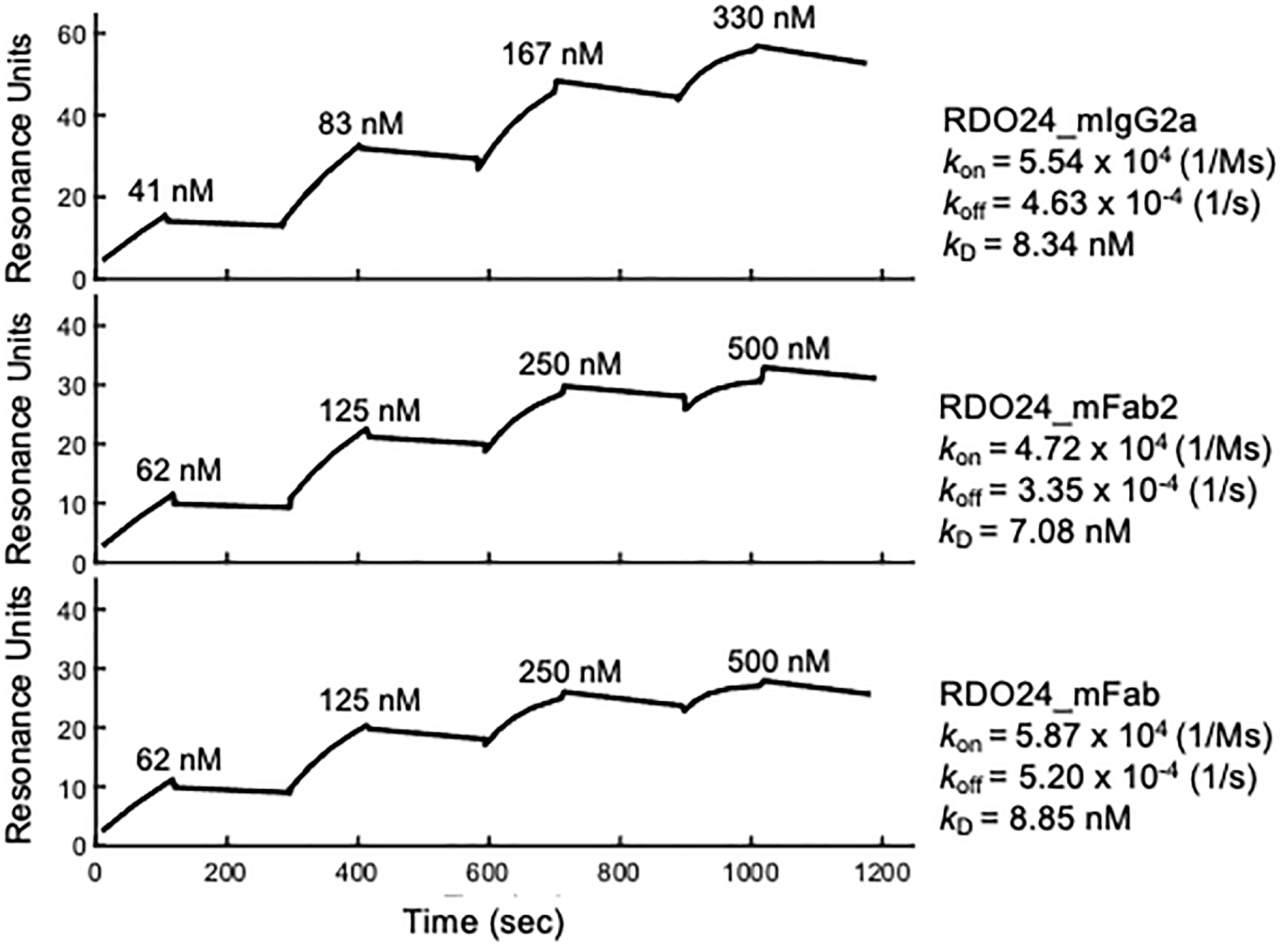
Binding kinetics of RDO24_mIgG2a, RDO24_mFab2 and RDO24_mFab. Human MET ECD-Fc was immobilized on the sensor chip, and binding of increasing concentrations of engineered antibodies was determined by surface plasmon resonance.

The binding kinetics of RDO24 molecules to recombinant human MET extracellular domain-fragment crystallizable region (ECD-Fc) were determined by means of Surface Plasmon Resonance (SPR) using BiacoreT100. The specificity of the interaction between RDO24 and hMET ECD-Fc was evaluated by single cycle kinetic analysis. As described in the “*Material and Methods*” section, the human MET ECD-Fc was directly immobilized on a CM5 sensor chip by amine coupling. Then, increasing concentrations of RDO24_mIgG2a, RDO24_mFab2, and RDO24_mFab were passed onto the sensor chip, and the kinetic of their interaction with MET was measured with respect to the antigen association (*k*_on_) and dissociation rate (*k*_off_), from which we calculated the dissociation constant (*k*_D_) of each construct toward the MET ECD-Fc target. The resulting SPR sensorgrams revealed a high affinity interaction between human MET ECD-Fc and RDO24_mIgG2a or RDO24_mFab or RDO24_mFab2, with *k*_D_ value of 8.34 nM, or 8.85 nM or 7.08 nM, respectively (**Figure 2**). Moreover, the kinetic profile of the tested fragments revealed a low dissociation rate supporting a highly stable binding with the MET receptor.

### RDO24 Cross-Reacts With Human, Rat and Mouse c-MET

**Figure 3:**
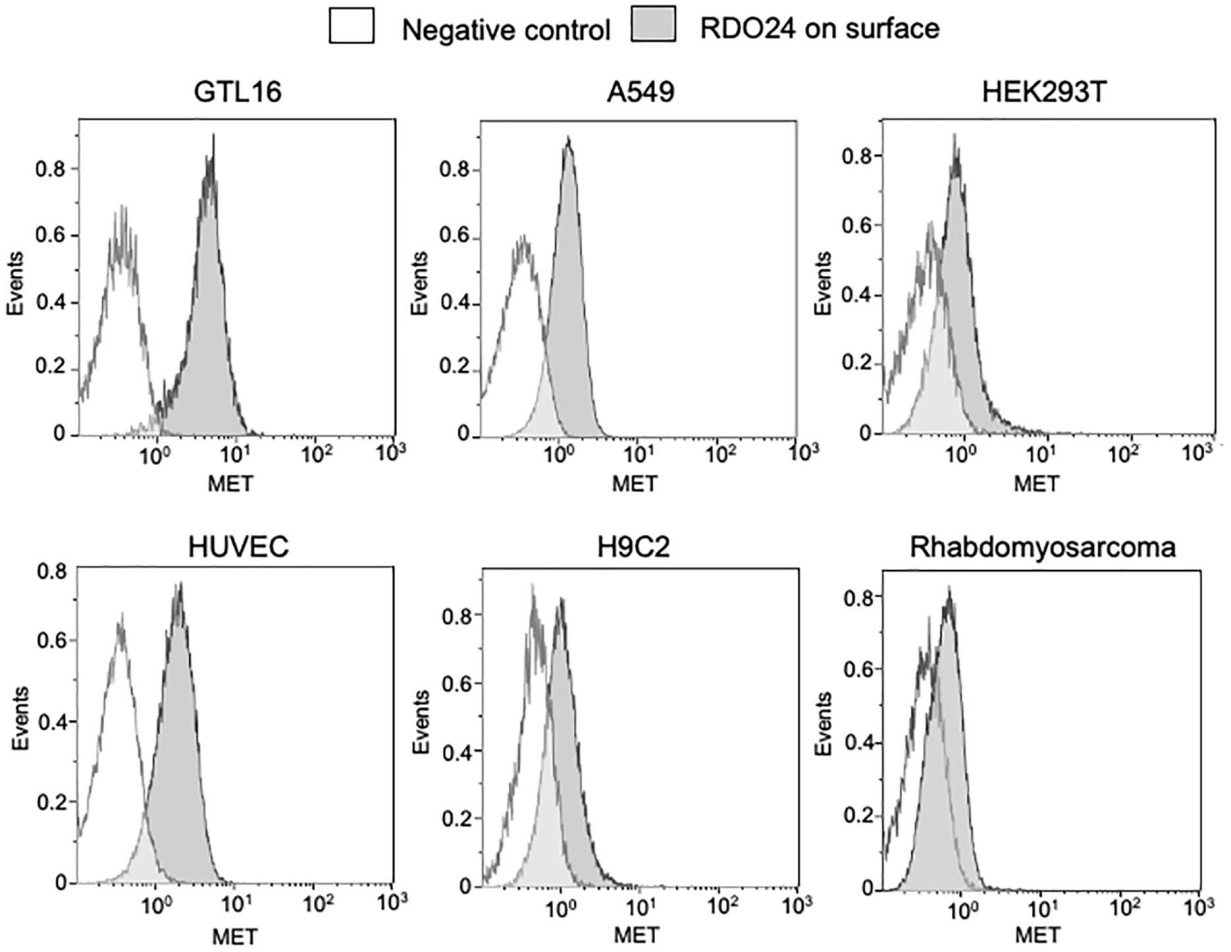
RDO24_mIgG2a cross-reacts with human, rat, and mouse MET receptor. MET surface protein levels were measured by flow cytometry in human GTL16, A549, HEK293T, and HUVEC, in rat H9c2, and in mouse rhabdomyosarcoma cells by using RDO24_mIgG2a labelled by PE-Cy7.

By using flow cytometry experimental approach, we demonstrated that RDO24 antibody binds the MET receptor of human, rat and mouse origin (**Figure 3**). RDO24 mAb recognized the surface MET protein expressed in different types of human cells: GTL16 gastric carcinoma, A549 lung adenocarcinoma, HEK293T human embryonic kidney cells, and HUVEC human umbilical vein endothelial cells (**Figure 3**). MAbs interacted also with the MET receptor of rat cardiomyoblast H9c2 and mouse rhabdomyosarcoma cell lines (**Figure 3**).

### Mapping of the RDO24 Epitope on the PSI Domain of MET Receptor

**Figure 4:**
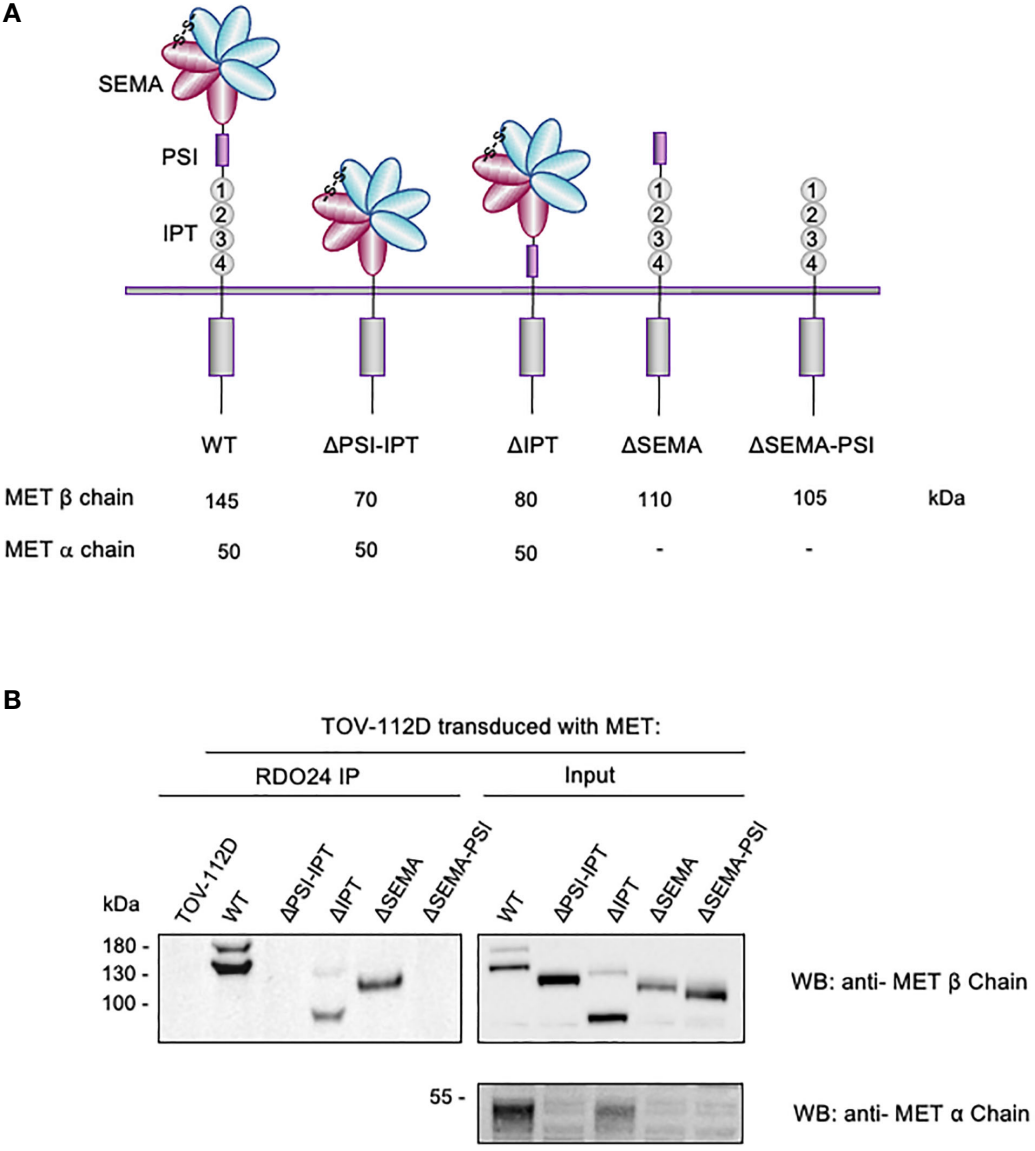
RDO24_IgG2a binding requires the PSI domain of MET receptor. Schematic representation of domain deletion constructs of MET **(A)**. TOV-112D cells were transduced with MET wild type (WT) or MET deletion constructs and immunoprecipitated with RDO24 mAb (left panel). A fraction of total lysates was used as the input control (right panel). Western blots were probed with antibodies against the intracellular (upper panels) and extracellular (lower panel) domains of MET **(B)**.

The extracellular region of the MET receptor consists of a semaphorin (SEMA) domain, a plexin semaphorin-integrin (PSI) domain (similar in structure to the plexins, semaphorins and integrins), and four immunoglobulin-like regions found in plexins and transcription factor (IPT) (MET WT in **Figure 4A**). The SEMA domain consists of a seven-blade β-propeller fold: the blades 1-4 comprise the MET α-chain while the blades 5-7 are part of the β-chain. The full-length single-chain MET precursor, in fact, is cleaved into two disulfide-linked chains of 50 kDa α- and 145 kDa β- subunits in the Golgi. The DO24 antibody was previously shown to bind the extracellular portion of MET β-chain (45), but its precise binding site was not determined. In order to precisely map the MET epitope recognized by RDO24 mAb, we used MET WT and different engineered mutant molecules constituted by deleted portions of the extracellular domains fused to transmembrane and intracellular domains of the receptor: MET ΔPSI-IPT, MET ΔIPT, MET ΔSEMA, MET ΔSEMA-PSI (**Figure 4A**). All proteins contain the endogenous leader sequence at the N-terminus. The constructs were transfected in TOV-112D cells, which do not express MET. As shown in **Figure 4B**, RDO24 mAb was able to immunoprecipitate full-length MET WT, resolved in the β-chain (145kDa) and α-chain (50kDa) under reducing conditions. Among the deleted proteins, RDO24 immunoprecipitated the MET ΔIPT (80 kDa β-chain and 50 kDa α-chain) and MET ΔSEMA (110 kDa βchain), but not the MET ΔPSI-IPT or MET ΔSEMA-PSI mutant proteins. The MET ΔPSI-IPT migrated as a 120 kDa single-chain protein, suggesting that it was not cleaved into the αβ heterodimer. These data indicate that RDO24 likely recognizes an epitope located in the PSI domain of the MET receptor.

### RDO24 Binds the ECD of MET and Not That of Closely Related RON and SEMA4D

**Figure 5:**
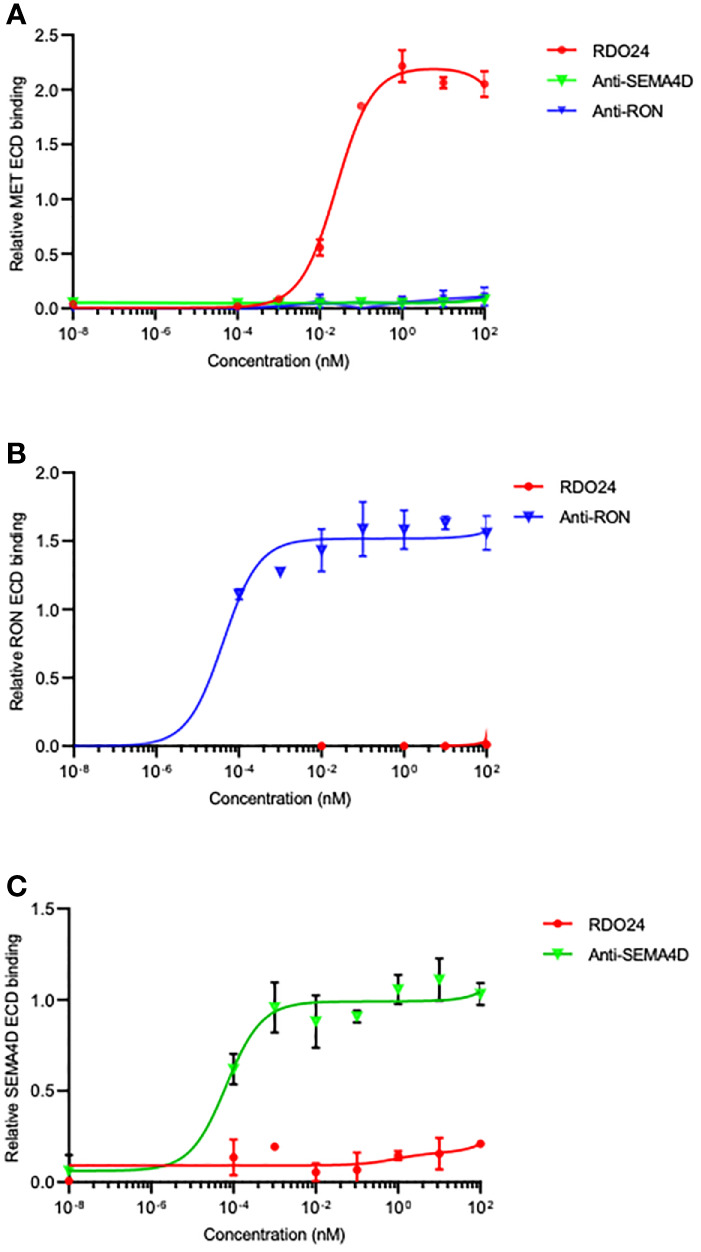
RDO24 mAb selectively binds the MET receptor. Binding of RDO24_mIgG2a (red line) to the extracellular domain (ECD)-fragment crystallizable region (Fc) of human MET **(A)**, RON **(B)**, and SEMA4D **(C)** was measured by enzyme-linked immunosorbent assay. The anti-RON and anti-SEMA4D antibodies were used as controls of respective ECD-Fc chimeras.

The PSI domain is structurally conserved in MET family members such as recepteur d’origine nantais (RON) (53) and Semaphorin4D (SEMA4D) (54). To evaluate whether RDO24 specifically binds to MET, we performed ELISA binding assays with MET, RON and SEMA4D ECD. We showed that RDO24 binds the ECD of MET (**Figure 5A**) and not that of closely related RON (**Figure 5B**) and SEMA4D (**Figure 5C**), indicating the selectivity of RDO24_mIgG2a against MET protein.

### RDO24 Docks Onto the PSI Domain of MET in Computational Modeling Analysis

**Figure 6:**
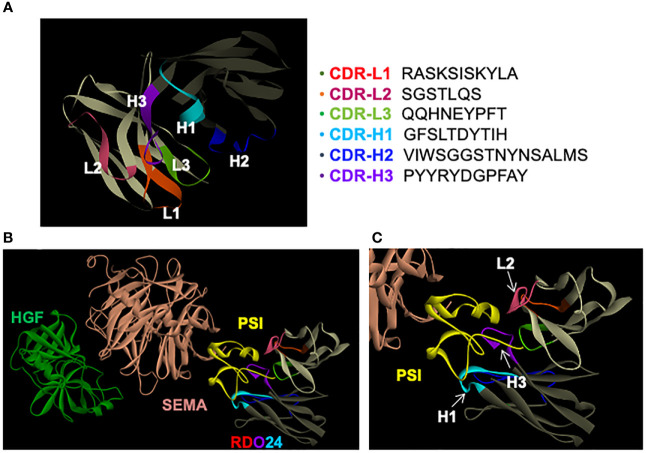
RDO24 mAb docks on the PSI domain of MET. A three-dimensional model of RDO24 variable fragment (Fv) was generated by Rosetta homology modeling. The complementarity determining regions (CDR) of the V_H_ and V_L_ domains are represented in colors. The framework regions of the V_H_ and V_L_ are shown in dark and light grey, respectively. The CDR sequences of RDO24 are represented according to the Clothia numbering scheme **(A)**. RDO24 was docked to MET (PDB accession: 1SHY) using the ZDOCK docking program. The figures were drawn with Discovery Studio Visualizer. The CDR of the V_H_ and V_L_ domains are shown in colors. The SEMA domain is shown in light pink, the PSI domain in yellow and HGF in green **(B)**; enlarged view in **(C)**.

A computational modeling was performed in order to dock RDO24 Fv fragments onto the MET structure available in the Protein Data Bank (PDB) online archive with the code 1SHY (55). We generated a three-dimensional structural model of Fv RDO24 using the Rosetta-based computational homology modeling technique (**Figure 6A**) (48). Next, we used the protein-protein docking program ZDOCK (49) to dock the Fv RDO24 on top of the 1SHY structure of MET. HGF binds the SEMA domain (light pink) of MET (**Figure 6B**). Superposition of RDO24 Fv fragments with MET showed that the CDR-H1, CDR-H3 and CDR-L2 strongly interact with PSI domain (yellow) of MET (**Figures 6B, C** **)**.

### RDO24_mIgG2a and RDO24_mFab2 Are Endowed With MET Agonistic Activity

**Figure 7:**
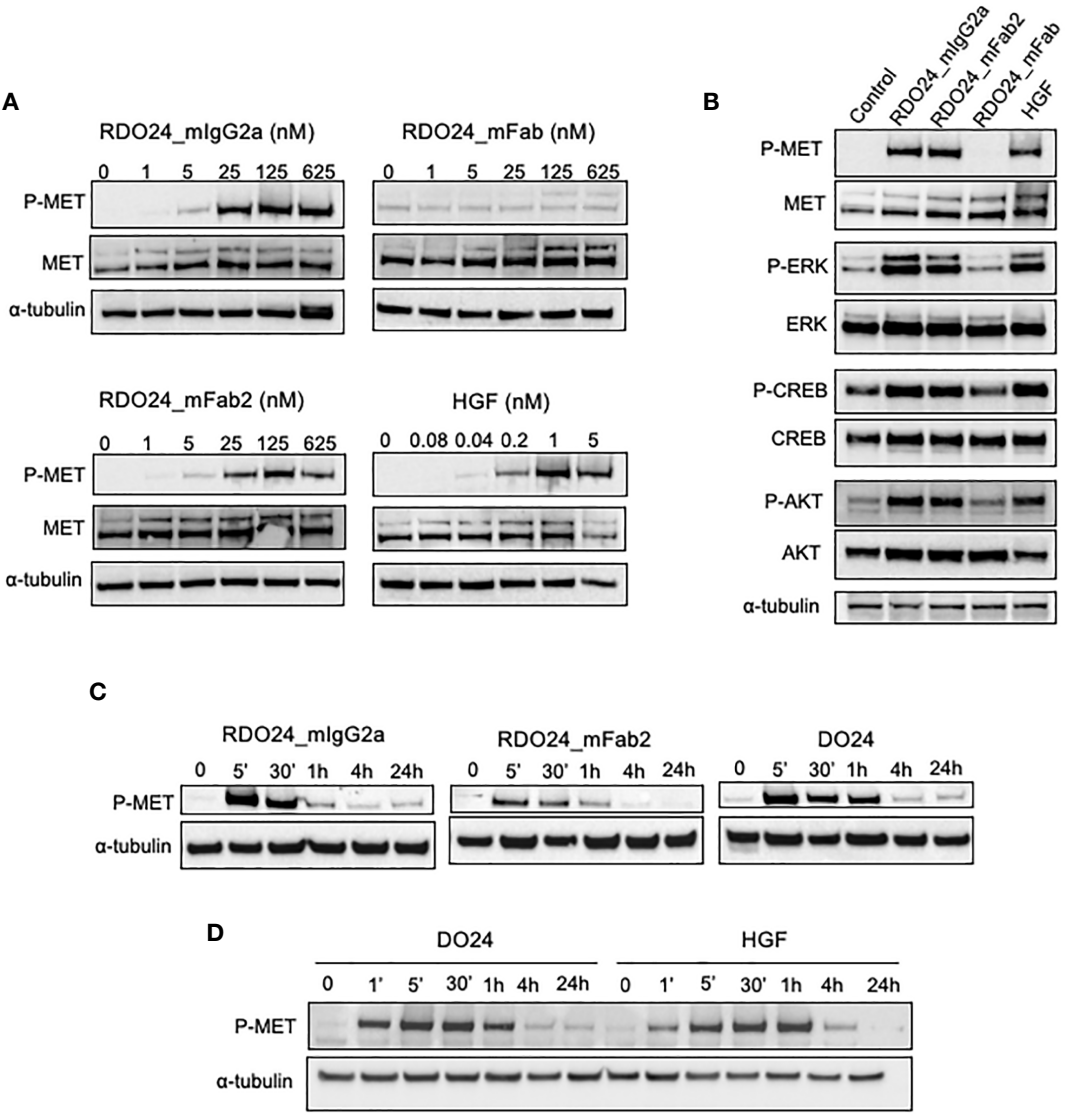
RDO24_mIgG2a and RDO24_mFab2, but not RDO24_mFab, induce phosphorylation of MET and stimulation of its main downstream pathways. Starved A549 cells were treated for 5’ with RDO24_mIgG2a, RDO24_mFab2, RDO24_mFab, and HGF. Protein phosphorylation was analyzed by western blotting using specific antibodies. Dose response analysis of MET phosphorylation at Y1234/1235 by recombinant molecules **(A)**. MAbs were used at 125 nM and HGF at 1 nM to compare phosphorylation of MET and downstream signaling proteins **(B)**. Time course analysis of MET phosphorylation at Y1234/1235 were performed using mAbs (RDO24 and the primitive antibody DO24) at 50 nM and HGF at 0.6 nM **(C, D)**. α-tubulin was used as loading control.

The DO24 mAb was previously shown to promote MET phosphorylation and therefore acts as an agonist of the receptor (46). This is likely due to the bivalent nature of the antibody, which enables the MET receptor dimerization, trans-phosphorylation, and activation of the downstream signaling. To verify this hypothesis the MET activation potency of bivalent (whole and mFab2) and the monovalent (mFab) molecules was assessed. A549 cells were treated with either molecule, lysed and analyzed in western blot (**Figure 7**). HGF was used as positive control. The RDO24_IgG2a and mFab2, but not the mFab, induced phosphorylation of MET at Y1234/1235 in a dose-dependent manner (**Figures 7A, B** **)**. Furthermore, RDO24_IgG2a and mFab2, but not the mFab, stimulated phosphorylation of ERK (extracellular signal regulated kinase) and AKT, the two main signaling pathways downstream MET, to a similar extent to that induced by HGF (**Figure 7B**). The downstream ERK effector, CREB (cAMP response element binding protein) transcription factor, was also phosphorylated after treatment with bivalent antibodies (**Figures 7B**). Time course experiments revealed that the bivalent RDO24 molecules showed MET phosphorylation responses that were comparable in time to the ones elicited by DO24 monoclonal antibody, from which they were derived, or by the natural HGF ligand (**Figures 7C, D** **)**. These data indicate that the RDO24_IgG2a and Fab2 fragment own agonistic properties for MET leading to stimulation of receptor and its downstream signaling pathways.

### RDO24_mIgG2a and RDO24_mFab2 Mimic the HGF-Mediated Biological Effects

**Figure 8:**
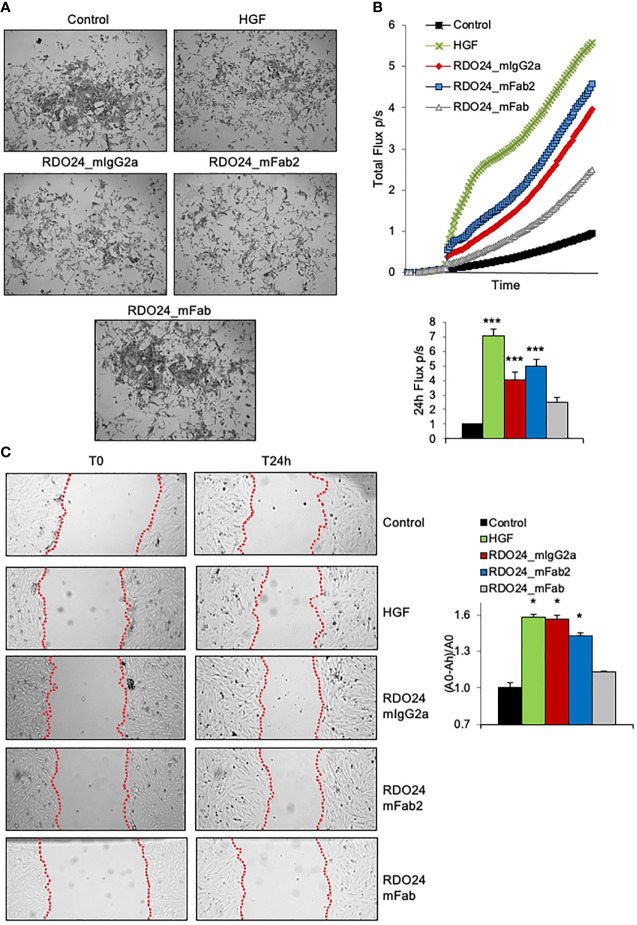
RDO24_mIgG2a and RDO24_mFab2, but not RDO24_mFab, trigger MET biological activity. HGF (0.5 nM) and mAbs (50 nM) were tested for scatter effect in epithelial MDCK cells **(A)**, X-Celligence cell migration assay in HPAF-II cells **(B)**, and wound-healing assay in H9c2 cardiomyoblasts cells **(C)**. Data analysis in **(B, C)** were obtained from three independent experiments. ***Pvalue < 0.001 significant *vs* Control; *Pvalue < 0.05 significant *vs* Control.

One of the unique biological effects induced by HGF in epithelial cells is cell-cell dissociation. In fact, HGF (also known as scatter factor) was originally discovered as a fibroblast-derived factor able to induce cell scattering (2). As shown in **Figure 8A**, incubation of MDCK epithelial cells with HGF induced a morphological change from epithelial colonies into scattered individual cells. Importantly, both the entire and the Fab2 mAbs, but not the Fab fragment, exerted a scattering effect on MDCK cells, comparable to that of HGF (**Figure 8A**). For cell motility assay, HPAF-II cells were treated with MET agonists and analyzed by the XCelligence Systems for 24 h. Activation of MET receptor by HGF induced strong cell migration as compared to untreated cells (**Figure 8B**). Both full-size mAb and Fab2 produced a significant biological effect over control, though at lower extent as compared to HGF (**Figure 8B**). In a ‘wound-healing’ assay, treatment with either bivalent molecule induced rat H9c2 cardiomyoblast cells to migrate and to cover the wounded area to the similar extent as HGF (**Figure 8C**). Overall, these data suggest that both entire and Fab2 mAbs mimic the migratory effects of HGF.

### RDO24 Delays the Onset of Clinical Symptoms in EAE Mouse Model *In Vivo*

**Figure 9:**
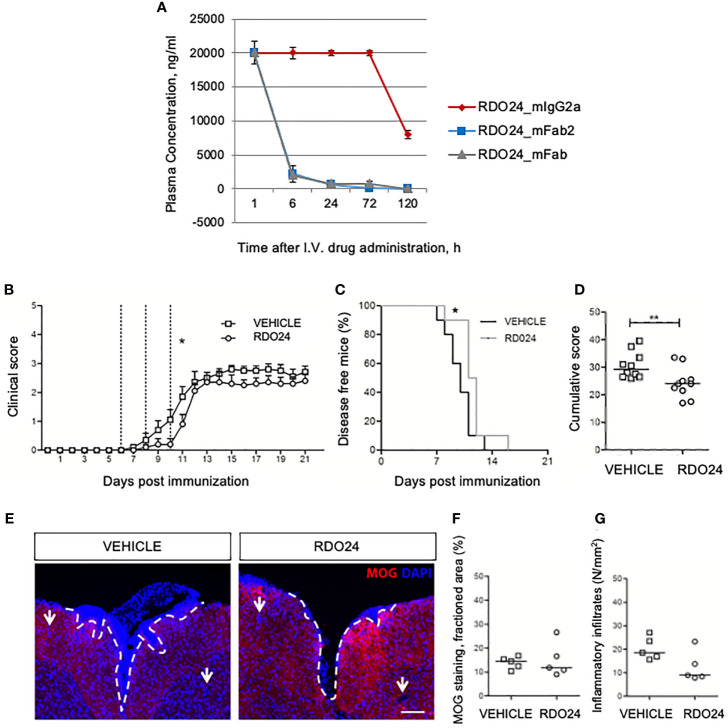
RDO24 administration delays the onset of EAE. RDO24_mIgG2a, RDO24_mFab2, and RDO24_mFab plasma concentration kinetics were evaluated by ELISA after intravenous (I.V.) delivery in mice **(A)**. The clinical course of EAE in RDO24 (n=10) and vehicle (n=10) –treated EAE mice was compared as mean clinical score **(B)**, percentage of disease free mice **(C)**, and cumulative **(D)** score. Dashed line indicates the days of treatment (6, 8, and 10 days post immunization). **(E)** Representative immunofluorescence images of coronal sections of spinal cord of vehicle (n=5) and RDO24 (n=5) -treated EAE mice stained with anti-MOG antibody (red). DAPI (blue) counterstains cell nuclei. Demyelination and perivascular inflammatory infiltrates were measured using respectively MOG fractioned area **(F)**, and DAPI+ perivascular inflammatory infiltrates density **(G)** in vehicle- and RDO24-treated EAE mice. White arrows indicate the perivascular inflammatory infiltrates. Calibration bars, 100µm. Two Way ANOVA, Bonferroni post-test **(B)**; Chi square test **(C)**; Mann-Whitney U test **(D, F, G)**. *Pvalue < 0.05 significant *vs* Vehicle; **Pvalue < 0.01 significant *vs* Vehicle.

To evaluate the stability of the new molecules in blood circulation *in vivo*, a single dose of RDO24_mIgG2a, RDO24_mFab2, and RDO24_mFab was injected intravenously and the plasma concentrations were determined by ELISA assay. As shown in **Figure 9A**, the full-size antibody was very stable whereas the Fab2 and Fab molecules were lost 6 hours after the injection. These data suggested that the full-size antibody was a more suitable molecule for *in vivo* treatments. To evaluate the effect of RDO24 on the onset of MOG_35–55_-induced EAE, immunized mice received the compound intravenously three times once every two days beginning from the 6^th^ dpi, when phenotypic EAE signs are not yet evident, but the immunization process has already occurred. RDO24 treatment was able to significantly delay the disease onset as reported by analysis of clinical score during days (**Figure 9B**) and percent of disease-free mice (**Figure 9C**). The cumulative score of RDO24-treated EAE mice was also significantly reduced in comparison to vehicle (**Figure 9D**). Mice were sacrificed in the 21^th^ dpi, and the spinal cords were stained with MOG antibody for the assessment of demyelination in the white matter (**Figure 9E**). No significant differences emerged in RDO24-treated EAE mice in comparison to vehicle concerning MOG-fractioned area (**Figure 9F**). However, RDO24 treatment caused a reducing trend in density of perivascular inflammatory infiltrates (**Figure 9G**), suggesting that a protective effect of RDO24 may result from its modulation of infiltrating inflammatory cells.

## Discussion

MET is a tyrosine kinase receptor that is physiologically activated by its unique HGF ligand. Activation of MET promotes cell proliferation and survival, is involved in tissue protection and repair, and represents an interesting target for regenerative medicine. Unfortunately, despite its therapeutic potential, HGF is a poor drug candidate. Exogenous HGF administered by intravenous injection is rapidly cleared by the liver *in vivo* (56, 57). Moreover, HGF is sequestered by the low affinity–high avidity sites widespread among the extracellular matrix proteoglycans (58–60). Additionally, as the endogenous HGF is post-translationally modified and correctly folded after its biosynthesis, the industrial production of bioactive HGF is hampered by the difficulty of producing a fully active molecule. Hence, the clinical use of HGF is limited and its substitution with molecules mimicking HGF, such as agonist monoclonal antibodies, is justified. Monoclonal antibodies are useful human therapeutics because of their specificity, affinity, and structure stability. Furthermore, they can easily be engineered *in vitro* using synthetic genes (61).

HGF is produced and secreted as an inactive single-chain pro-HGF form. Proteolytic cleavage is required to obtain the disulfide-bond linked two-chains HGF, which is the active form able to activate MET. MET can also be activated by bypassing the proteolytic processing of single chain pro HGF through an allosteric mechanism involving peptides. In particular, the so-called peptide V8 was found to bind the single-chain pro-HGF resulting in a similar conformation of two-chain HGF observed in the activation cleavage pathway, representing a new approach for MET signaling activation (62). Another strategy to mimic HGF activity is the generation of artificial MET bivalent macrocyclic peptides, covalently bound by linkers such as Polyethylene Glycol (PEG) or carbon chains. Bivalent macrocyclic peptides can dimerize and activate MET depending on the linker length and show a full agonism towards MET, thus mimicking HGF activation in a similar extent (63). A further approach is the engineering of HGF fragments. HGF is composed of an N-terminal hairpin domain, four Kringle domains, and a C-terminal serine protease homology domain (64). Even if the N-terminal and first kringle fragment (NK1) occurs as a natural variant able to activate MET receptor, it is limited by its low stability and weak agonist activity. NK1 mutants were thus engineered to achieve a better stability and higher agonist potency and disulfide linked NK1 homo dimers showed a similar agonistic activity to that of full-length HGF (65). Furthermore, a modern approach is the targeting of MET receptor with antibodies. Herein, we describe the engineering and characterization of a monoclonal murine antibody acting as MET receptor agonist that might be useful for regenerative medicine.

For this purpose, we generated the new recombinant RDO24_mIgG2a and mFab2 fragment by genetic engineering. The RDO24_ mIgG2a and mFab2 were created to produce bivalent molecules with a unique variable portion (VH-VL) interacting with the cognate MET antigen. These molecules contain two antigen binding portions (VLCL and VHCH1) linked together by a disulphide bond. The presence of two identical monovalent binding sites may give to the antibody molecules the agonistic property. For this reason, we tested whether recombinant full-length and Fab2 molecule mimic the biological effects exerted by the natural HGF ligand in molecular, cellular, and biological assays. There are many advantages of using Fab2 fragment antibodies instead of whole IgG antibodies. Fab2 fragment antibodies do not have Fc portions, thus they eliminate non-specific binding between Fc portions of antibodies and Fc receptors on cells (such as macrophages, dendritic cells, neutrophils, NK cells and B cells). Indeed, for a stimulating antibody to be used in regenerative medicine it is an advantage in avoiding the Fc-mediated functions such as antibody-dependent cell-mediated cytotoxicity or complement-dependent cytotoxicity. Another advantage is that Fab2 fragments penetrate tissues more efficiently than whole IgG antibodies due to their smaller size. Last, but not least the smaller size of fragments may permit cheaper, faster production in microbial systems. On the other hand, the smaller size of Fab2 fragments may give rise to shorter half-life and increase renal clearance, representing a limitation for the development in therapy.

HGF binding upon MET leads to its dimerization, which is mandatory for its activation. Activation occurs by trans-phosphorylation of the two catalytic tyrosines Y1234-Y1235 belonging to the tyrosine kinase domain. Two docking tyrosines (Y1349-Y1356) of the C-terminal domain are subsequently phosphorylated and signal proteins, such as Gab1 (GRB2-associated-binding protein 1), Src, PI3K (phosphoinositide-3-kinase), GRB2 (Growth factor receptor-bound protein 2), PLCγ (phospholipase c gamma) and STAT3 (signal transducer and activator of transcription 3), containing a binding site for Src-homology-2 (SH2) or a phosphorylation domain, are recruited (44). These events lead to the activation of MET effectors, such as AKT and RAS-MAPK (mitogen-activated protein kinase) pathway, which are required for MET biological functions. We found that stimulation of A549 cells with bivalent whole antibody and Fab2 fragment induced MET phosphorylation at Y1234/1235 and, consequently, enhanced the levels of P-AKT and P-ERK, which are the main signaling downstream of MET. Furthermore, the transcription factor CREB, an effector of ERK, was also phosphorylated. Stimulation with monovalent Fab did not exert the same results. Thus, a bivalency-driven mechanism of action confers agonistic properties to RDO24_mIgG2a and mFab2 for MET activation and induction of its downstream signaling pathways. In line with this, the bivalent but not the monovalent mAbs evoke MET triggered biological effects, including cell scattering, migration, and wound healing. Our mAb cross reacts with mouse and rat MET. This represents an important requirement for pre-clinical models of regenerative medicine, requiring the antibody to be employed on rodent tissues and cells.

To test the potentially promising perspective of RDO24 agonist molecules to reach the preclinical phase, we performed *in vivo* analysis in mice. Unfortunately, the Fab2 fragment showed a short half-life in blood circulation. Thus, we considered the more stable full-size antibody for preclinical studies. We used the EAE mouse model of MS, a disease characterized by leucocytes infiltration and accumulation in the central nervous system and local destruction of myelin and neurons. Previous work showed that HGF exerts anti-inflammatory and immunosuppressive functions, showing efficacy in the mitigation of EAE (38, 66). Our results *in vivo* suggest that RDO24 is a functional HGF mimetic and may be beneficial in inflammatory diseases. However, there is an important caveat for pharmacological intervention at the level of MET activation to protect from inflammation and promote tissue regeneration. In fact, MET is involved in cancer progression, and some tumors are driven by MET alterations, such as amplification and overexpression (22). Further studies are necessary to ultimately elucidate the window of therapeutic benefit, without inducing adverse effects.

The MET extracellular domain is composed by three types of structural domains: SEMA, PSI, and IPT (1–4). The SEMA domain is structured as a seven-bladed propeller, whose blades 1-4 comprise the MET α-chain while blades 5-7 belong to the β-chain. HGF consists of two α and β subunits linked by a disulphide bond, as mentioned in the introduction. The HGF α-chain contains the high-affinity MET binding site (8) and interacts with SEMA domain (67) and also with IPT3,4 regions (13). The cleaved β-chain of HGF was crystallized together with MET SEMA-PSI domains (55) and interacts with low affinity with the bottom face of blades 2 and 3 of SEMA domain. While the HGF α chain is sufficient for MET binding, the cleaved β chain is necessary for MET activation (55, 68). The MET PSI domain does not interact with HGF. Although its function is not known, circumstantial evidence suggests that it may work as an hinge and likely orients the adjacent domains SEMA and IPT for proper ligand binding (69, 70).

We previously showed that DO24 binds to the extracellular portion of MET β-chain and does not compete with HGF binding (46). A SPR analysis revealed a high affinity and stable interaction of our recombinant full-lenght mAb and its fragments, with human MET ECD-Fc, with an equilibrium kD in the low nanomolar range, and a kinetic profile typical of highly stable protein-protein interactions. Furthermore, deletion mapping studies showed that the binding of RDO24 to the MET ECD was abolished by deletion of the PSI domain. The involvement of PSI domain in its binding to MET was confirmed by computational modeling. The PSI domain is structurally conserved in MET family members such as RON (53) and SEMA4D (54). Our mAb recognizes the PSI domain of the MET ECD but not the same domain of RON and SEMA4D receptors, belonging to MET family, thus suggesting a selectivity towards MET. Different monoclonal antibodies have been raised to the extracellular domain of MET (see Prat et al., 2014 for a review) (71). The bivalent antibodies directed to the SEMA domain usually behave as agonists, as the SEMA contains the low and high affinity binding sites for HGF (11) and is critical for receptor dimerization and activation (12). The bivalent agonist antibody (5D5) against the SEMA domain competes with HGFα binding site and was converted to a monovalent one-armed human IgG1 format to become antagonist (72). The DN30 antibody, endowed with partial agonist activity, proved to be a potent antagonist when engineered to a monovalent Fab (73). The MET epitope recognized by DN30 is within the IPT4 domain (74), a region included in one of the identified HGF binding sites (domains IPT3 IPT4) (13). Notably, the “antagonist” mechanism of DN30 is associated to induction of proteolytic cleavage of MET ECD (receptor “shedding”) followed by proteasome-mediated receptor degradation (73). In both cases, the conversion of a bivalent antibody into a monovalent form has been used to minimize the agonist activity. The role of PSI domain in the regulation of MET is still unknown. Recently, an antagonistic antibody specific to the PSI domain of MET, IRCR201, has been reported (75). IRCR201 is a human IgG1 bivalent antibody, which induces rapid depletion of MET protein *via* the lysosomal degradation pathway and inhibits tumor growth *in vitro* and *in vivo*. Our bivalent anti-PSI antibody is a mouse IgG2a and is endowed with potent agonist activity. It is plausible that the IgG architecture contributes to the antagonist/agonist activity of the antibodies (76). Future work will show whether constructing a human bivalent and/or monovalent one-armed IgG1 RDO24 antibody may be effective to create an inhibitory anti-MET antibody for cancer therapy.

## Data Availability Statement

The original contributions presented in the study are included in the article/supplementary material. Further inquiries can be directed to the corresponding author.

## Author Contributions

CD contributed to figures realization, performed the ELISA assays and the docking study, and wrote the manuscript with TC and SG. SG and AV performed the experiments and realized the figures. EV cloned DO24 RNA hybridoma. CB and FZ realized MET mutant constructs and their transduction in TOV112D cells. FM performed treatments and analysis on EAE mice. EC performed the flow cytometry assays. RM and DMF performed SPR-based analysis. AB and PMC revised the manuscript and TC engineered the antibodies and wrote and revised the manuscript. All authors contributed to the article and approved the submitted version.

## Funding

This work was supported by Fondazione Italiana Sclerosi Multipla (FISM), AIRC 5permille Program N.21719, AIRC IG N. 23820 (grant number 2017/R/9) and by Italian Ministry of Health “Ricerca Corrente 2021”.

## Conflict of Interest

EV and PMC own shares in Metis BeCorp. RM and DMF are co-founders of the academic spin-off IXTAL, in which they hold shares (www.ixtal.it).

The remaining authors declare that the research was conducted in the absence of any commercial or financial relationships that could be construed as a potential conflict of interest.

## Publisher’s Note

All claims expressed in this article are solely those of the authors and do not necessarily represent those of their affiliated organizations, or those of the publisher, the editors and the reviewers. Any product that may be evaluated in this article, or claim that may be made by its manufacturer, is not guaranteed or endorsed by the publisher.

## Abbreviations

CH, heavy chain; CL, light chain; CDR, complementary determining regions; CREB, cAMP response elementbinding protein; EAE, experimental autoimmune encephalomyelitis; ECD, extracellular domain; ERK, extracellular signal regulated kinase; Gab1, GRB2-associated-binding protein 1; GRB2, growth factor receptor-bound protein 2; HGF, hepatocyte growth factor; IgG2a, immunoglobulin 2a; IPT, immunoglobulin-like plexins-transcription factor; mAb, monoclonal antibody; MAPK, mitogen-activated protein kinase; MOG_35–55_, myelin oligodendrocyte glycoprotein; MS, multiple sclerosis; PDB, protein data bank; PI3K, phosphoinositide-3-kinase; PLCγ, phospholipase c gamma; PSI, plexin-semaphorin-integrin; RON, recepteurd’origine nantaise; RT-PCR, reverse transcriptase-polymerase chain reaction; SEMA, semaphorin; SEMA4D, semaphorin 4D; SH2, Src-homology-2; SPR, surface plasmon resonance; STAT3, signal transducer and activator of transcription 3; VL, light chain variable domain; VH domain, heavy chain variable domain; WT, wild-type.

## References

[1] Nakamura T Nishizawa T Hagiya M Seki T Shimonishi M Sugimura A. Molecular Cloning and Expression of Human Hepatocyte Growth Factor. Nature (1989) 342:440–3. doi: 10.1038/342440a0 2531289

[2] Stoker M Gherardi E Perryman M Gray J. Scatter Factor Is a Fibroblast-Derived Modulator of Epithelial Cell Mobility. Nature (1987) 327:239–42. doi: 10.1038/327239a0 2952888

[3] Naldini L Tamagnone L Vigna E Sachs M Hartmann G Birchmeier W. Extracellular Proteolytic Cleavage by Urokinase Is Required for Activation of Hepatocyte Growth Factor/Scatter Factor. EMBO J (1992) 11:4825–33. doi: 10.1002/j.1460-2075.1992.tb05588.x PMC556958 1334458

[4] Miyazawa K. Hepatocyte Growth Factor Activator (HGFA): A Serine Protease That Links Tissue Injury to Activation of Hepatocyte Growth Factor. FEBS J (2010) 277:2208–14. doi: 10.1111/j.1742-4658.2010.07637.x 20402766

[5] Shimomurasj T Kondos J Ochiais M Nakas D Miyazawall K. Activation of the Zymogen of Hepatocyte Growth Factor Activator by Thrombin. J Biol Chem (1993) 268:22927–32. doi: 10.1016/S0021-9258(18)41615-8 8226803

[6] Naldini L Weidner KM Vigna E Gaudino G Bardelli A Ponzetto C. Scatter Factor and Hepatocyte Growth Factor Are Indistinguishable Ligands for the MET Receptor. EMBO J (1991) 10:2867–78. doi: 10.1002/j.1460-2075.1991.tb07836.x PMC452997 1655405

[7] Bottaro D Rubin J Faletto D Chan A Kmiecik T Vande Woude G. Identification of the Hepatocyte Growth Factor Receptor as the C-Met Proto-Oncogene Product. Science (1991) 251:802–4. doi: 10.1126/science.1846706 1846706

[8] Lokker NA Mark MR Luis EA Bennett GL Robbins KA Baker JB. Structure-Function Analysis of Hepatocyte Growth Factor: Identification of Variants That Lack Mitogenic Activity Yet Retain High Affinity Receptor Binding. EMBO J (1992) 11:2503–10. doi: 10.1002/j.1460-2075.1992.tb05315.x PMC556725 1321034

[9] Kirchhofer D Yao D Peek XM Eigenbrot C Lipari MT Billeci KL. Structural and Functional Basis of the Serine Protease-Like Hepatocyte Growth Factor β-Chain in Met Binding and Signaling. J Biol Chem (2004) 279:39915–24. doi: 10.1074/jbc.M404795200 15218027

[10] Komada M Hatsuzawa K Shibamoto S Ito F Nakayama K Kitamura N. Proteolytic Processing of the Hepatocyte Growth Factor/Scatter Factor Receptor by Furin. FEBS Lett (1993) 328:25–9. doi: 10.1016/0014-5793(93)80958-w 8344430

[11] Gherardi E Youles ME Miguel RN Blundell TL Iamele L Gough J. Functional Map and Domain Structure of MET, the Product of the C-Met Protooncogene and Receptor for Hepatocyte Growth Factor/Scatter Factor. Proc Natl Acad Sci USA (2003) 100:12039–44. doi: 10.1073/pnas.2034936100 PMC218709 14528000

[12] Kong-Beltran M Stamos J Wickramasinghe D. The Sema Domain of Met Is Necessary for Receptor Dimerization and Activation. Cancer Cell (2004) 6:75–84. doi: 10.1016/J.CCR.2004.06.013 15261143

[13] Basilico C Arnesano A Galluzzo M Comoglio PM Michieli P. A High Affinity Hepatocyte Growth Factor-Binding Site in the Immunoglobulin-Like Region of Met. J Biol Chem (2008) 283:21267. doi: 10.1074/JBC.M800727200 18495663 PMC2475716

[14] Gentile A Trusolino L Comoglio PM. The Met Tyrosine Kinase Receptor in Development and Cancer. Cancer Metastasis Rev (2008) 27:85–94. doi: 10.1007/s10555-007-9107-6 18175071

[15] Maina F Casagranda F Audero E Simeone A Comoglio PM Klein R. Uncoupling of Grb2 From the Met Receptor *In Vivo* Reveals Complex Roles in Muscle Development. Cell (1996) 87:531–42. doi: 10.1016/s0092-8674(00)81372-0 8898205

[16] Bladt F Riethmacher D Isenmann S Aguzzi A Birchmeier C. Essential Role for the C-Met Receptor in the Migration of Myogenic Precursor Cells Into the Limb Bud. Nature (1995) 376:768–71. doi: 10.1038/376768a0 7651534

[17] Maina F Klein R. Hepatocyte Growth Factor, A Versatile Signal for Developing Neurons. Nat Neurosci Publ (1999) 2:213–7. doi: 10.1038/6310 10195212

[18] Uehara Y Minowa O Mori C Shiota K Kuno J Noda T. Placental Defect and Embryonic Lethality in Mice Lacking Hepatocyte Growth Factor/Scatter Factor. Nature (1995) 373:702–5. doi: 10.1038/373702a0 7854453

[19] Schmidt C Bladt F Goedecke S Brinkmann V Zschiesche W Sharpe M. Scatter Factor/Hepatocyte Growth Factor Is Essential for Liver Development. Nature (1995) 373:699–702. doi: 10.1038/373699a0 7854452

[20] Birchmeier C Birchmeier W Gherardi E Vande Woude GF. Met, Metastasis, Motility and More. Nat Rev Mol Cell Biol (2003) 4:915–25. doi: 10.1038/nrm1261 14685170

[21] Bussolino F. Hepatocyte Growth Factor Is a Potent Angiogenic Factor Which Stimulates Endothelial Cell Motility and Growth. J Cell Biol (1992) 119:629–41. doi: 10.1083/jcb.119.3.629 PMC2289675 1383237

[22] Trusolino L Bertotti A Comoglio PM. MET Signalling: Principles and Functions in Development, Organ Regeneration and Cancer. Nat Rev Mol Cell Biol (2010) 11:834–48. doi: 10.1038/nrm3012 21102609

[23] Sakai K Aoki S Matsumoto K. Hepatocyte Growth Factor and Met in Drug Discovery. J Biochem (2015) 157:271–84. doi: 10.1093/jb/mvv027 25770121

[24] Desole C Gallo S Vitacolonna A Montarolo F Bertolotto A Vivien D. From Brain Development to Neurological Disorders. Front Cell Dev Biol (2021) 9:683609. doi: 10.3389/fcell.2021.683609 PMC8220160 34179015

[25] Gallo S Gatti S Sala V Albano R Costelli P Casanova E. Agonist Antibodies Activating the Met Receptor Protect Cardiomyoblasts From Cobalt Chloride-Induced Apoptosis and Autophagy. Cell Death Dis (2014) 5:e1185–5. doi: 10.1038/cddis.2014.155 PMC4001309 24743740

[26] Gallo S Spilinga M Albano R Ferrauto G Di Gregorio E Casanova E. Activation of the Met Receptor Attenuates Doxorubicin-Induced Cardiotoxicity *In Vivo* and *In Vitro* . Br J Pharmacol (2020) 177:3107–22. doi: 10.1111/bph.15039 PMC7280013 32133617

[27] Urbanek K Rota M Cascapera S Bearzi C Nascimbene A De Angelis A. Cardiac Stem Cells Possess Growth Factor-Receptor Systems That After Activation Regenerate the Infarcted Myocardium, Improving Ventricular Function and Long-Term Survival. Circ Res (2005) 97:663–73. doi: 10.1161/01.RES.0000183733.53101.11 16141414

[28] Gallo S Sala V Gatti S Crepaldi T. Cellular and Molecular Mechanisms of HGF/Met in the Cardiovascular System. Clin Sci (2015) 129:1173–93. doi: 10.1042/CS20150502 26561593

[29] Huh CG Factor VM Sánchez A Uchida K Conner EA Thorgeirsson SS. Hepatocyte Growth Factor/C-Met Signaling Pathway Is Required for Efficient Liver Regeneration and Repair. Proc Natl Acad Sci USA (2004) 101:4477–82. doi: 10.1073/pnas.0306068101 PMC384772 15070743

[30] Giebeler A Boekschoten MV Klein C Borowiak M Birchmeier C Gassler N. C-Met Confers Protection Against Chronic Liver Tissue Damage and Fibrosis Progression After Bile Duct Ligation in Mice. Gastroenterology (2009) 137:297–308, 308.e1–4. doi: 10.1053/j.gastro.2009.01.068 19208365

[31] Marquardt JU Seo D Gómez-Quiroz LE Uchida K Gillen MC Kitade M. Loss of C-Met Accelerates Development of Liver Fibrosis in Response to CCl 4 Exposure Through Deregulation of Multiple Molecular Pathways. Biochim Biophys Acta Mol Basis Dis (2012) 1822:942–51. doi: 10.1016/j.bbadis.2012.02.012 PMC3338880 22386877

[32] Zhou D Tan RJ Lin L Zhou L Liu Y. Activation of Hepatocyte Growth Factor Receptor, C-Met, in Renal Tubules Is Required for Renoprotection After Acute Kidney Injury. Kidney Int (2013) 84:509–20. doi: 10.1038/ki.2013.102 PMC3758808 23715119

[33] Stella GM Gentile A Baderacchi A Meloni F Milan M Benvenuti S. Ockham’s Razor for the MET-Driven Invasive Growth Linking Idiopathic Pulmonary Fibrosis and Cancer. J Transl Med (2016) 14:1–12. doi: 10.1186/s12967-016-1008-4 27590450 PMC5010719

[34] Ware LB Matthay MA. Keratinocyte and Hepatocyte Growth Factors in the Lung: Roles in Lung Development, Inflammation, and Repair. Am J Physiol Lung Cell Mol Physiol (2002) 282:L924–40. doi: 10.1152/ajplung.00439.2001 11943656

[35] Nakamura T Sakai K Nakamura T Matsumoto K. Hepatocyte Growth Factor Twenty Years on: Much More Than a Growth Factor. J Gastroenterol Hepatol (2011) 26:188–202. doi: 10.1111/j.1440-1746.2010.06549.x 21199531

[36] Yasuda H Imai E Shiota A Fujise N Morinaga T Higashio K. Antifibrogenic Effect of a Deletion Variant of Hepatocyte Growth Factor on Liver Fibrosis in Rats. Hepatology (1996) 24:636–42. doi: 10.1002/hep.510240328 8781336

[37] Mizuno S Kurosawa T Matsumoto K Mizuno-Horikawa Y Okamoto M Nakamura T. Hepatocyte Growth Factor Prevents Renal Fibrosis and Dysfunction in a Mouse Model of Chronic Renal Disease. J Clin Invest (1998) 101:1827–34. doi: 10.1172/JCI1709 PMC508767 9576745

[38] Bai L Lennon DP Caplan AI DeChant A Hecker J Kranso J. Hepatocyte Growth Factor Mediates Mesenchymal Stem Cell–Induced Recovery in Multiple Sclerosis Models. Nat Neurosci (2012) 15:862–70. doi: 10.1038/nn.3109 PMC3427471 22610068

[39] Jeffers M Schmidt L Nakaigawa N Webb CP Weirich G Kishida T. Activating Mutations for the Met Tyrosine Kinase Receptor in Human Cancer. Proc Natl Acad Sci USA (1997) 94:11445–50. doi: 10.1073/pnas.94.21.11445 PMC23495 9326629

[40] Comoglio PM Giordano S Trusolino L. Drug Development of MET Inhibitors: Targeting Oncogene Addiction and Expedience. Nat Rev Drug Discov (2008) 7:504–16. doi: 10.1038/nrd2530 18511928

[41] Gherardi E Birchmeier W Birchmeier C Vande Woude G. Targeting MET in Cancer: Rationale and Progress. Nat Rev Cancer (2012) 12:89–103. doi: 10.1038/nrc3205 22270953

[42] Zarnegar R. Regulation of HGF and HGFR Gene Expression. EXS (1995) 74:33–49. doi: 10.1007/978-3-0348-9070-0_3 8527900

[43] Nakamura T Matsumoto K Kiritoshi A Tano Y Nakamura T. Induction of Hepatocyte Growth Factor in Fibroblasts by Tumor-Derived Factors Affects Invasive Growth of Tumor Cells: *In Vitro* Analysis of Tumor-Stromal Interactions. Cancer Res (1997) 57:3305–13. 9242465

[44] Comoglio PM Trusolino L Boccaccio C. Known and Novel Roles of the MET Oncogene in Cancer: A Coherent Approach to Targeted Therapy. Nat Rev Cancer (2018) 1:341–58. doi: 10.1038/s41568-018-0002-y 29674709

[45] Prat M Crepaldi T Gandino L Giordano S Longati P Comoglio P. C-Terminal Truncated Forms of Met, the Hepatocyte Growth Factor Receptor. Mol Cell Biol (1991) 11:5954–62. doi: 10.1128/MCB.11.12.5954.Updated PMC361753 1944272

[46] Prat M Crepaldi T Pennacchietti S Bussolino F Comoglio PM. Agonistic Monoclonal Antibodies Against the Met Receptor Dissect the Biological Responses to HGF. J Cell Sci (1998) 111:237–47. doi: 10.1242/jcs.111.2.237 9405310

[47] Vigna E Naldini L. Lentiviral Vectors: Excellent Tools for Experimental Gene Transfer and Promising Candidates for Gene Therapy. J Gene Med (2000) 2:308–16. doi: 10.1002/1521-2254(200009/10)2:5<308::aid-jgm131>3.0.co;2-3 11045424

[48] Weitzner BD Jeliazkov JR Lyskov S Marze N Kuroda D Frick R. Modeling and Docking of Antibody Structures With Rosetta. Nat Protoc (2017) 12:401–16. doi: 10.1038/nprot.2016.180 PMC5739521 28125104

[49] Pierce BG Wiehe K Hwang H Kim BH Vreven T Weng Z. ZDOCK Server: Interactive Docking Prediction of Protein-Protein Complexes and Symmetric Multimers. Bioinformatics (2014) 30:1771–3. doi: 10.1093/bioinformatics/btu097 PMC4058926 24532726

[50] Montarolo F Raffaele C Perga S Martire S Finardi A Furlan R. Effects of Isoxazolo-Pyridinone 7e, A Potent Activator of the Nurr1 Signaling Pathway, on Experimental Autoimmune Encephalomyelitis in Mice. PloS One (2014) 9:e108791. doi: 10.1371/journal.pone.0108791 25265488 PMC4181297

[51] Montarolo F Perga S Martire S Bertolotto A. Nurr1 Reduction Influences the Onset of Chronic EAE in Mice. Inflamm Res (2015) 64:841–4. doi: 10.1007/s00011-015-0871-4 26337347

[52] Furlan C Montarolo F Di Gregorio E Parolisi R Atlante S Buffo A. Analysis of the Gadolinium Retention in the Experimental Autoimmune Encephalomyelitis (EAE) Murine Model of Multiple Sclerosis. J Trace Elem Med Biol (2021) 68:126831. doi: 10.1016/j.jtemb.2021.126831 34364067

[53] Chao KL Tsai IW Chen C Herzberg O. Crystal Structure of the Sema-PSI Extracellular Domain of Human RON Receptor Tyrosine Kinase. PloS One (2012) 7:e41912. doi: 10.1371/journal.pone.0041912 22848655 PMC3405059

[54] Kozlov G Perreault A Schrag JD Park M Cygler M Gehring K. Insights Into Function of PSI Domains From Structure of the Met Receptor PSI Domain. Biochem Biophys Res Commun (2004) 321:234–40. doi: 10.1016/j.bbrc.2004.06.132 15358240

[55] Stamos J Lazarus RA Yao X Kirchhofer D Wiesmann C. Crystal Structure of the HGF β-Chain in Complex With the Sema Domain of the Met Receptor. EMBO J (2004) 23:2325–35. doi: 10.1038/sj.emboj.7600243 PMC423285 15167892

[56] Liu KX Kato Y Narukawa M Kim DC Hanano M Higuchi O. Importance of the Liver in Plasma Clearance of Hepatocyte Growth Factors in Rats. Am J Physiol (1992) 263:G642–9. doi: 10.1152/ajpgi.1992.263.5.G642 1443139

[57] Masumoto A Yamamoto N. Sequestration of a Hepatocyte Growth Factor in Extracellular Matrix in Normal Adult Rat Liver. Biochem Biophys Res Commun (1991) 174:90–5. doi: 10.1016/0006-291X(91)90489-T 1824922

[58] Lyon M Deakin JA Rahmoune H Fernig DG Nakamura T Gallagher JT. Hepatocyte Growth Factor/Scatter Factor Binds With High Affinity to Dermatan Sulfate. J Biol Chem (1998) 273:271–8. doi: 10.1074/jbc.273.1.271 9417075

[59] Kemp LE Mulloy B Gherardi E. Signalling by HGF/SF and Met: The Role of Heparan Sulphate Co-Receptors. Biochem Soc Trans (2006) 34:414–7. doi: 10.1042/BST0340414 16709175

[60] Rubin JS Day RM Breckenridge D Atabey N Taylor WG Stahl SJ. Dissociation of Heparan Sulfate and Receptor Binding Domains of Hepatocyte Growth Factor Reveals That Heparan Sulfate-C-Met Interaction Facilitates Signaling. J Biol Chem (2001) 276:32977–83. doi: 10.1074/jbc.M105486200 11435444

[61] De Samblanx H Schrijvers D. Monoclonal Antibodies. In: ESMO Handb. Princ. Transl. Res. Abingdon: CRC Press (2007). p. 115–20. doi: 10.3109/9781420044386-16

[62] Landgraf KE Santell L Billeci KL Quan C Young JC Maun HR. Allosteric Peptide Activators of Pro-Hepatocyte Growth Factor Stimulate Met Signaling. J Biol Chem (2010) 285:40362–72. doi: 10.1074/jbc.M110.179721 PMC3001016 20937841

[63] Ito K Sakai K Suzuki Y Ozawa N Hatta T Natsume T. Artificial Human Met Agonists Based on Macrocycle Scaffolds. Nat Commun (2015) 6:6373. doi: 10.1038/ncomms7373 25758345 PMC4382702

[64] Donate LE Srinivasan N Sowdhamini R Gherardi E Blundell TL Aparicio S. Molecular Evolution and Domain Structure of Plasminogen-Related Growth Factors (HGF/SF and HGF1/MSP). Protein Sci (1994) 3:2378–94. doi: 10.1002/pro.5560031222 PMC2142779 7756992

[65] Jones DS Tsai P-C Cochran JR. Engineering Hepatocyte Growth Factor Fragments With High Stability and Activity as Met Receptor Agonists and Antagonists. Proc Natl Acad Sci USA (2011) 108:13035–40. doi: 10.1073/pnas.1102561108 PMC3156163 21788476

[66] Benkhoucha M Santiago-Raber ML Schneiter G Chofflon M Funakoshi H Nakamura T. Hepatocyte Growth Factor Inhibits CNS Autoimmunity by Inducing Tolerogenic Dendritic Cells and CD25+Foxp3+ Regulatory T Cells. Proc Natl Acad Sci USA (2010) 107:6424–9. doi: 10.1073/pnas.0912437107 PMC2851995 20332205

[67] Gherardi E Sandin S Petoukhov MV Finch J Youles ME Ofverstedt L-G. Structural Basis of Hepatocyte Growth Factor/Scatter Factor and MET Signalling. Proc Natl Acad Sci (2006) 103:4046–51. doi: 10.1073/pnas.0509040103 PMC1449643 16537482

[68] Matsumoto K Kataoka H Date K Nakamura T. Cooperative Interaction Between α- and β-Chains of Hepatocyte Growth Factor on C-Met Receptor Confers Ligand-Induced Receptor Tyrosine Phosphorylation and Multiple Biological Responses. J Biol Chem (1998) 273:22913–20. doi: 10.1074/jbc.273.36.22913 9722511

[69] Niemann HH Jäger V Butler PJG van den Heuvel J Schmidt S Ferraris D. Structure of the Human Receptor Tyrosine Kinase Met in Complex With the Listeria Invasion Protein InlB. Cell (2007) 130:235–46. doi: 10.1016/j.cell.2007.05.037 17662939

[70] Basilico C Hultberg A Blanchetot C De Jonge N Festjens E Hanssens V. Four Individually Druggable MET Hotspots Mediate HGF-Driven Tumor Progression. J Clin Invest (2014) 124:3172–86. doi: 10.1172/JCI72316 PMC4071368 24865428

[71] Prat M Oltolina F Basilico C. Monoclonal Antibodies Against the MET/HGF Receptor and Its Ligand: Multitask Tools With Applications From Basic Research to Therapy. Biomedicines (2014) 2:359–83. doi: 10.3390/biomedicines2040359 PMC5344273 28548076

[72] Wilson TR Fridlyand J Yan Y Penuel E Burton L Chan E. Widespread Potential for Growth-Factor-Driven Resistance to Anticancer Kinase Inhibitors. Nature (2012) 487:505–9. doi: 10.1038/nature11249 PMC3724525 22763448

[73] Pacchiana G Chiriaco C Stella MC Petronzelli F De Santis R Galluzzo M. Monovalency Unleashes the Full Therapeutic Potential of the DN-30 Anti-Met Antibody. J Biol Chem (2010) 285:36149–57. doi: 10.1074/jbc.M110.134031 PMC2975237 20833723

[74] Vigna E Chiriaco C Cignetto S Fontani L Basilico C Petronzelli F. Inhibition of Ligand-Independent Constitutive Activation of the Met Oncogenic Receptor by the Engineered Chemically-Modified Antibody DN30. Mol Oncol (2015) 9:1760–72. doi: 10.1016/j.molonc.2015.05.007 PMC5528712 26119717

[75] Park H Kim D Kim E Sa J Lee H Yu S. Tumor Inhibitory Effect of IRCR201, a Novel Cross-Reactive C-Met Antibody Targeting the PSI Domain. Int J Mol Sci (2017) 18:1968. doi: 10.3390/ijms18091968 PMC5618617 28902178

[76] Maun HR Vij R Walters BT Morando A Jackman JK Wu P. Bivalent Antibody Pliers Inhibit β-Tryptase by an Allosteric Mechanism Dependent on the IgG Hinge. Nat Commun (2020) 11:6435. doi: 10.1038/s41467-020-20143-x 33353951 PMC7755903

